# Haem toxicity provides a competitive advantage to the clinically relevant *Staphylococcus aureus* small colony variant phenotype

**DOI:** 10.1099/mic.0.001044

**Published:** 2021-04-19

**Authors:** Brittany E. Herrin, Shariful Islam, Kaitlin N. Rentschler, Lauren H. Pert, Stephanie P. Kopanski, Catherine A. Wakeman

**Affiliations:** ^1^​ Department of Biological Sciences, Texas Tech University, Lubbock, TX, USA; ^†^​ Present address: Department of Biology, Indiana University, Bloomington, IN, USA

**Keywords:** *Staphylococcus aureus*, SCV, antibiotic tolerance, haem toxicity, respiration, Small colony variants

## Abstract

Microorganisms encounter toxicities inside the host. Many pathogens exist as subpopulations to maximize survivability. Subpopulations of *Staphylococcus aureus* include antibiotic-tolerant small colony variants (SCVs). These mutants often emerge following antibiotic treatment but can be present in infections prior to antibiotic exposure. We hypothesize that haem toxicity in the host selects for respiration-deficient *S. aureus* SCVs in the absence of antibiotics. We demonstrate that some but not all respiration-deficient SCV phenotypes are more protective than the haem detoxification system against transient haem exposure, indicating that haem toxicity in the host may contribute to the dominance of menaquinone-deficient and haem-deficient SCVs prior to antibiotic treatment.

## Abbreviations

c.f.u., colony forming units; PBS, Phosphate buffered saline; SCV, small colony variant; TSA, Tryptic soy agar; TSB, Tryptic soy broth; WT, Wild-type.

## Introduction

*Staphylococcus aureus* is a common human commensal, infecting approximately one-third of the human population, primarily colonizing the nasopharynx [1]. When released from this region, *S. aureus* can disseminate to various sites in the body to cause a range of infections, such as endocarditis, bacteraemia and osteomyelitis [2]. While in the host, *S. aureus* encounters a range of selective pressures. *S. aureus* adopts several mechanisms to survive and persist in the host environment in response to these pressures. One such adaptation is the small colony variant (SCV) subpopulation [2–4]. The SCV of *S. aureus* has several phenotypic differences from its wild-type (WT) counterparts, such as slow growth rate, lack of carotenoid pigmentation and reduced haemolysis [5]. Collectively, these traits result in suppression of several virulence factors, yet this phenotype is often observed in chronic infections, such as cystic fibrosis and osteomyelitis [6–8]. As an apparent trade-off to this reduction in traditional virulence factors, SCVs do exhibit increased adhesion [9] as well as the ability to survive intracellularly in host cells [10], which may contribute to their emergence in chronic infections. Additionally, it has been previously demonstrated that the SCV phenotype can be enriched by exposure to antimicrobials produced by *Pseudomonas aeruginosa *, host-derived antimicrobial peptides or aminoglycoside antibiotics [11–14]. It is the antibiotic tolerance of SCVs that is typically thought to be the selective pressure contributing to their infective potential [15]. Numerous mutations can result in the SCV phenotype [2]. While thymidine-deficient SCVs are predominant in cystic fibrosis infections, respiration-deficient SCV mutants lacking either menaquinone or haem biosynthesis are among the most common clinical isolates from osteomyelitis or device-related infections [16]. Both osteomyelitis and device-related infections often are associated with microbial dissemination through the bloodstream [17].

In *S. aureus *, haem is essential in both aerobic and anaerobic respiration, as it serves as a cofactor in cytochromes. Additionally, this molecule serves as a common iron source within the host environment that tends to be depleted of free iron. Therefore, *S. aureus* undergoes metabolic effort to scavenge exogenous haem that is contained in host red blood cells [18, 19]. Paradoxically, excess haem is toxic to the bacteria, which is why the HrtAB haem detoxification system is expressed by *S. aureus* in environments rich in haem [20]. This system specifically acts to efflux excess haem [21, 22] and has been shown to be expressed in systemic infections, indicating that the environment of the bloodstream can induce haem toxicity in invading pathogens [23]. In addition to the haem efflux pump, it has been shown that SCVs lacking menaquinone production are intrinsically resistant to haem toxicity, even in strains lacking the HrtAB haem detoxification pump [24]. This resistance was attributed to a decrease in haem-associated superoxide radical production [24].

We hypothesize that selection for the SCV phenotype during infection can be mediated by transient exposure to toxic levels of haem encountered in the host bloodstream, such as when released by a cut or wound in the nasopharynx, even in the absence of the previously cited pressures. This phenomenon could explain the finding that the SCV phenotype appears to be a ‘natural’ part of the *S. aureus* infectious cycle, appearing in infections sometimes even in the absence of prior antibiotic selection [2, 25]. The data presented herein demonstrate that menaquinone deficiency has a greater contribution than the well-characterized HrtAB system to the survival of *S. aureus* in the presence of transient exposure to toxic haem levels.

## Methods

### Chemicals

Hemin was purchased from Alfa Aesar. All other chemicals were purchased from Fisher Scientific.

### Bacterial strains

All experiments were performed in the *S. aureus* clinical isolate Newman [26] and mutants derived in the Newman parental background. The Δ*hrtB* mutant lacking the haem detoxification system was previously characterized [20, 27]. This specific mutant was selected rather than the Δ*hrtA* mutant due to the pleiotropic effects of having the dysregulated permease function [27]. The Δ*menB* SCV lacking the production of menaquinone and the Δ*hrtB*Δ*menB* SCV lacking both menaquinone production and the haem detoxification system were previously described [20, 24]. The Δ*hemB* SCV lacking endogenous haem production and the Δ*cydB*Δ*qoxB* mutants were previously described [28].

### Haem exposure assays

Strains were streaked for isolation on tryptic soy agar (TSA) and incubated at 37 °C overnight. Replicates were grown in 150 µl tryptic soy broth (TSB) in a 96-well round-bottom plate, overnight at 37 °C with shaking at 180 rpm. In the morning, cells were diluted 1:10 in fresh TSB and allowed to grow for 1 h in 96-well round-bottom plates. These cells were then normalized to identical optical densities at 600 nm (OD_600_ of approximately 0.1) before haem treatment. Hemin solutions were freshly prepared immediately prior to use in every experiment in 0.1 M NaOH. Once a 10 mM haem stock solution was freshly suspended, the haem solutions were diluted to the appropriate test concentrations in TSB in a 96-well round-bottom plate, and 5 µl of normalized cells was added to each test condition. These samples were incubated with and without haem, shaking at 37 °C for 1 h. Each treatment was then serially diluted using PBS and spotted onto TSA plates for enumeration of colony-forming units (c.f.u.). Colonies were counted after 1–2 days of growth at 37 °C, graphed in Excel and analysed using a one-way ANOVA.

Co-culture haem exposure assays were performed in the same basic manner as described above with the exception that immediately prior to haem exposure, equivalent cell numbers of SCV colonies were mixed with the parental respiring counterpart. Differential c.f.u. counting of SCV and respiring colonies were performed via visual identification due to the clear phenotypic differences of the colony types.

## Results and discussion

### Menaquinone deficiency is more protective than the haem detoxification system in the presence of transient haem exposure

It has been demonstrated previously that the HrtAB haem detoxification system is robustly induced by haem treatment and protects against haem toxicity [20, 29]. These previous studies, which typically have relied on growth curve/optical density analysis, have demonstrated HrtAB-mediated protective effects as early as 2 h following haem exposure [20, 29]. These effects may begin even earlier within the growth curve of *S. aureus* but were previously not apparent due to the lack of sensitivity in optical density-based microbial growth measurements. Therefore, we sought to overcome these limitations by measuring growth and death rates at early time points in the growth curve using c.f.u. values to assess viable cell counts.

Because the HrtAB system requires time for induction at both the mRNA and protein levels following haem exposure [20, 29], we hypothesized that this transporter may not be fully effective at protecting cells against transient haem exposure, which has been defined in this study as exposures lasting less than 1 h. On the other hand, the protective effects of menaquinone deficiency against haem toxicity are intrinsic to these cells due to a reduction in the generation of haem-related superoxide radicals [24]. Therefore, we hypothesize that menaquinone-deficient mutants will have a selective advantage over respiring strains of *S. aureus* following haem exposure despite the existence of the intrinsic haem detoxification system.

**Fig. 1.**
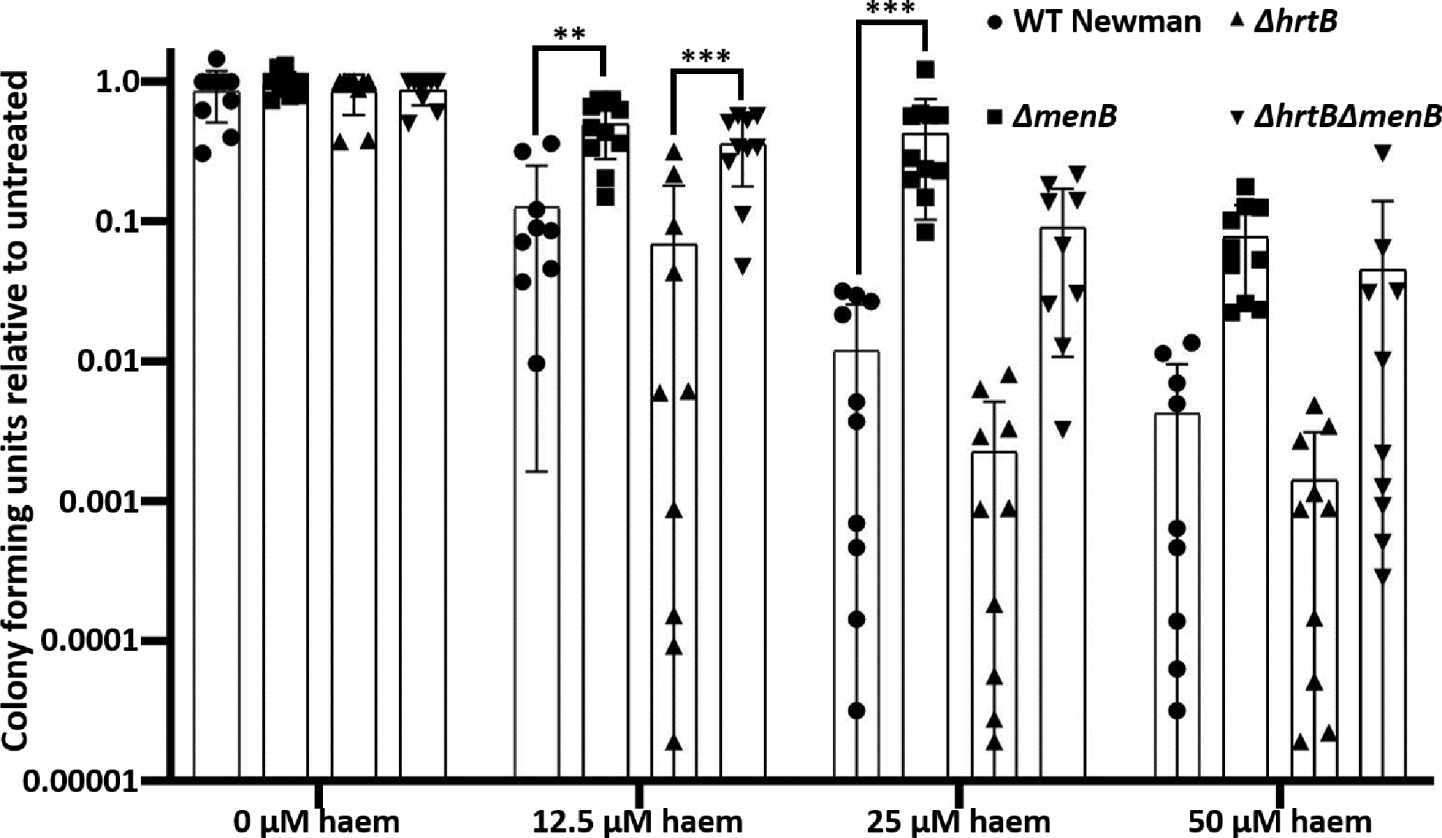
Menaquinone deficiency protects against transient haem stress more robustly than the HrtAB detoxification system. Bacterial strains of *S. aureus* were exposed to a range of haem concentrations 1 h prior to plating to determine viable cell counts, demonstrated by the number of c.f.u. present. While *ΔhrtB* strains appeared slightly though not statistically more sensitive to haem toxicity than strains containing the detoxification pump (e.g. WT Newman or *ΔmenB*), the protective effect of the *ΔmenB* mutation was statistically significant and even more striking than the protective effect of the HrtAB detoxification system. Graphs depict an average of values collected on at least ten independent days. On each independent day the experiment was performed in triplicate. Error bars depict standard deviation. **P<0.01 and ***P<0.001 as determined by a one-way ANOVA and Tukey’s post-hoc test.

Menaquinone-deficient mutants and their respiring parental strains either lacking or possessing the HrtAB haem detoxification pump were exposed to a range of haem levels for 1 h prior to serial dilution and c.f.u. plating to determine the number of viable cells remaining. Because healthy erythrocytes contain ~21 µM free haem [30] and lysed or traumatized blood cells are likely to contain even higher levels, we decided to use a transient haem treatment range of 12.5–50 µM. During this transient haem exposure, increasing levels of haem were shown to significantly reduce the number of viable cells remaining in a titratable manner for all strains tested (Fig. 1). The slight trend for higher survival of WT Newman over Δ*hrtB* Newman indicated that the HrtAB detoxification system is at least partially functional at these early time points of haem exposure and, as expected, this phenomenon becomes more pronounced with increasing haem concentrations. Interestingly, the protective effect of the menaquinone deficiency in either Δ*menB* or Δ*menB*Δ*hrtB* was even more striking and independent of the presence of the HrtAB efflux pump, indicating that either the loss of respiration and/or the absence of menaquinone has a more powerful protective effect than expression of the haem detoxification system after 1 h of haem exposure.

### Menaquinone-deficient SCVs are more protected from haem toxicity than other SCVs

**Fig. 2.**
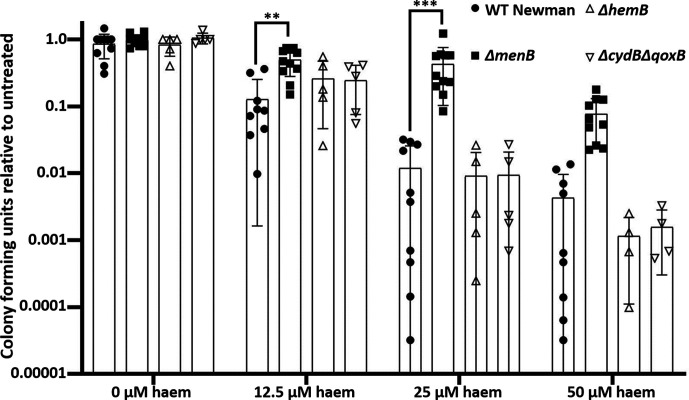
Menaquinone-deficient SCVs are more protected from transient haem stress than other SCVs. Bacterial strains of *S. aureus* were exposed to a range of haem concentrations 1 h prior to plating to determine viable cell counts, demonstrated by the number of c.f.u. The *ΔmenB* mutant was the only SCV tested exhibiting greater survival than the WT parental strain. Graphs depict an average of values collected on at least five independent days. On each independent day, the experiment was performed in triplicate. Error bars depict standard deviation. **P<0.01 and ***P<0.001 as determined by a one-way ANOVA and Tukey’s post-hoc test.

Mutations in the menaquinone biosynthesis pathway, endogenous haem biosynthesis pathway or pathways for cytochrome production all eliminate the ability of *S. aureus* to respire and thus confer the SCV phenotype [2, 28]. Menaquinone deficiency has been shown to provide protection under conditions of haem stress [24], and we sought to determine if all respiration-deficient SCV genotypes provide the same protection. We demonstrate that in haem concentrations exceeding 25 µM, the menaquinone-deficient mutant, *∆menB*, has significantly better survivorship than both the WT Newman and other SCV strains (Fig. 2). This indicated that it was not simply general loss of respiration that results in the protective effect but more specifically menaquinone deficiency. We hypothesize that this phenomenon could contribute to the observation that menaquinone mutants are particularly common in the clinic even though numerous SCV-inducing mutations can yield antibiotic tolerance and other metabolic features favouring persistence during infection [4, 31].

### Both menaquinone- and haem-deficient SCVs exhibit a competitive advantage over respiring *S. aureus* in mixed cultures

Even in mono-species populations of bacteria, subpopulations with altered gene expression exist [25, 32, 33]. The SCV phenotype in *S. aureus* in many ways mimics some of the phenotypic characteristics of persister cells, such as slow growth and resistance to antibiotics, and both states are naturally occurring [4, 25, 32, 34, 35]. To better mimic natural conditions in which the SCVs are a subpopulation present among respiring cells, we created co-cultures of WT and SCV strains to determine if the decreased haem susceptibility of menaquinone deficiency creates a competitive advantage for these cells. While we hypothesized that the *∆menB* strain would have a selective advantage over WT, we knew that the menaquinone crossfeeding found to occur in co-culture [7] may reverse the protection observed in mono-culture.

**Fig. 3.**
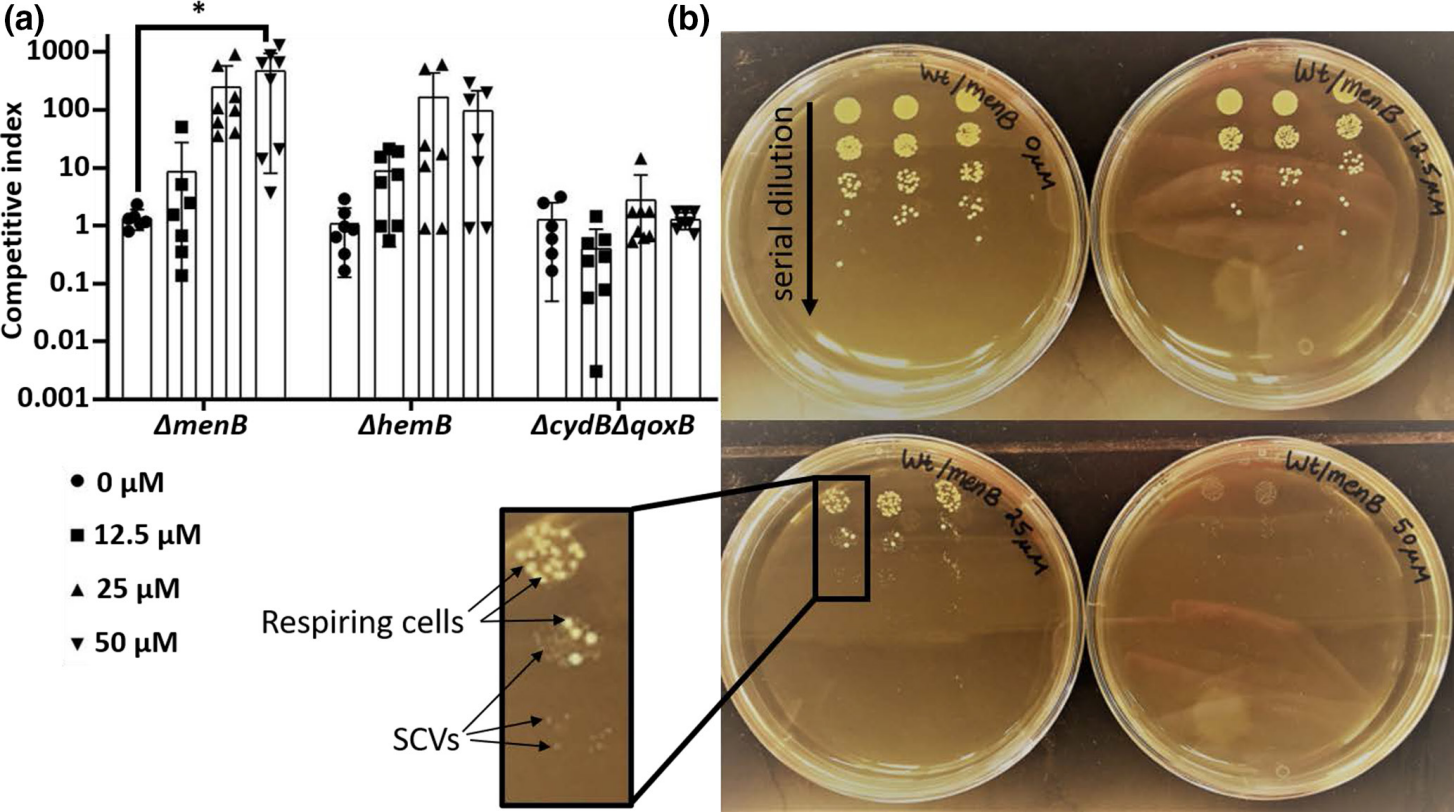
The SCV phenotype provides a competitive advantage in the presence of transient haem stress. (a) WT and SCV (either *ΔmenB*, *ΔhemB* or *ΔcydBΔqoxB*) were mixed in a 1:1 ratio and exposed to a range of haem concentrations for 1 h prior to plating to determine viable cell counts demonstrated by the number of c.f.u. The number of SCVs versus the number of respiring colonies (large colonies) was assessed, revealing that both menaquinone- and haem-deficient mutants exhibited a competitive advantage over WT in the presence of haem stress at levels exceeding 25 µM. Graphs depict an average of values collected on at least three independent days, which were performed in biological triplicates on each day. Error bars depict standard deviation. *P<0.05 as determined by a one-way ANOVA and Tukey’s post-hoc test.** (b**) Images of representative findings from viable cell counts derived from mixed cultures. After serial dilutions were performed, SCVs were easily distinguished from respiring colonies based on colony size (see inset).

After exposure to transient haem stress, the menaquinone mutants exhibited a robust competitive advantage over the parental strain as expected, especially at higher haem concentrations (Fig. 3a, b). Interestingly, survival of the *∆hemB* mutants also trended towards an increase in the co-culture condition, while the Δ*cydB*Δ*qoxB* mutants experienced the same amount of death as the respiring parental strain (Fig. 3a). This intermediate protective effect in the haem-deficient mutant may be attributed to the lower level of endogenous haem, which may also contribute to some level of haem toxicity and may also explain the identification of haem-deficient SCVs during certain *S. aureus* infections.

## Conclusions

The data presented herein demonstrate that the clinically relevant menaquinone-deficient SCV experiences a competitive advantage over respiring *S. aureus* when exposed to transient haem toxicity representative of conditions which may be experienced upon entry into the bloodstream prior to dissemination to sites of infection. Additionally, in mixed culture, this competitive advantage extends to clinically relevant haem-deficient SCV strains. This feature of SCVs may synergize with other SCV-associated behaviours to enable early colonization of infectious sites prior to antibiotic exposure. For example, the increased adhesive properties of *S. aureus* SCVs [9] may promote SCV colonization of distal body sites once disseminated via the bloodstream. In general, *S. aureus* SCVs are a threat to human health due to their propensity to emerge in various infectious niches and their ability to tolerate antibiotic treatment [4]. Therefore, efforts are currently being made to enable their rapid detection in infections [13, 36]. Similarly, efforts must be made to determine the most effective treatment strategies for this otherwise persistent cell type [13]. With combined early detection and effective treatment of SCVs, many morbidities associated with persistent *S. aureus* infections may be circumvented.

## References

[1] Gorwitz RJ, Kruszon-Moran D, McAllister SK, McQuillan G, McDougal LK (2008). Changes in the prevalence of nasal colonization with *Staphylococcus aureus* in the United States, 2001-2004. J Infect Dis.

[2] Proctor RA, von Eiff C, Kahl BC, Becker K, McNamara P (2006). Small colony variants: a pathogenic form of bacteria that facilitates persistent and recurrent infections. Nat Rev Microbiol.

[3] Bui LMG, Kidd SP (2015). A full genomic characterization of the development of a stable small colony variant cell-type by a clinical *Staphylococcus aureus* strain. Infect Genet Evol.

[4] Proctor RA, Kriegeskorte A, Kahl BC, Becker K, Löffler B (2014). *Staphylococcus aureus* small colony variants (SCVs): a road map for the metabolic pathways involved in persistent infections. Front Cell Infect Microbiol.

[5] Proctor RA, Balwit JM, Vesga O (1994). Variant subpopulations of Staphylococcus aureus as cause of persistent and recurrent infections. Infect Agents Dis.

[6] Proctor RA, van Langevelde P, Kristjansson M, Maslow JN, Arbeit RD (1995). Persistent and relapsing infections associated with small-colony variants of *Staphylococcus aureus*. Clin Infect Dis.

[7] Hammer ND, Cassat JE, Noto MJ, Lojek LJ, Chadha AD (2014). Inter- and intraspecies metabolite exchange promotes virulence of antibiotic-resistant Staphylococcus aureus. Cell Host Microbe.

[8] Sifri CD, Baresch-Bernal A, Calderwood SB, von Eiff C (2006). Virulence of *Staphylococcus aureus* small colony variants in the Caenorhabditis elegans infection model. Infect Immun.

[9] Vaudaux P, Francois P, Bisognano C, Kelley WL, Lew DP (2002). Increased expression of clumping factor and fibronectin-binding proteins by hemB mutants of *Staphylococcus aureus* expressing small colony variant phenotypes. Infect Immun.

[10] Vesga O, Groeschel MC, Otten MF, Brar DW, Vann JM (1996). Staphylococcus aureus small colony variants are induced by the endothelial cell intracellular milieu. J Infect Dis.

[11] Baishya J, Wakeman CA (2019). Selective pressures during chronic infection drive microbial competition and cooperation. NPJ Biofilms Microbiomes.

[12] Hoffman LR, Déziel E, D'Argenio DA, Lépine F, Emerson J (2006). Selection for *Staphylococcus aureus* small-colony variants due to growth in the presence of Pseudomonas aeruginosa. Proc Natl Acad Sci U S A.

[13] Kahl BC, Becker K, Löffler B (2016). Clinical significance and pathogenesis of staphylococcal small colony variants in persistent infections. Clin Microbiol Rev.

[14] Gläser R, Becker K, von Eiff C, Meyer-Hoffert U, Harder J (2014). Decreased susceptibility of *Staphylococcus aureus* small-colony variants toward human antimicrobial peptides. J Invest Dermatol.

[15] von Eiff C, Bettin D, Proctor RA, Rolauffs B, Lindner N (1997). Recovery of small colony variants of *Staphylococcus aureus* following gentamicin bead placement for osteomyelitis. Clin Infect Dis.

[16] Besier S, Smaczny C, von Mallinckrodt C, Krahl A, Ackermann H (2007). Prevalence and clinical significance of *Staphylococcus aureus* small-colony variants in cystic fibrosis lung disease. J Clin Microbiol.

[17] Kavanagh N, Ryan EJ, Widaa A, Sexton G, Fennell J (2018). *Staphylococcal osteomyelitis*: disease progression, treatment challenges, and future directions. Clin Microbiol Rev.

[18] Cassat JE, Skaar EP (2013). Iron in infection and immunity. Cell Host Microbe.

[19] Skaar EP, Humayun M, Bae T, DeBord KL, Schneewind O (2004). Iron-source preference of *Staphylococcus aureus* infections. Science.

[20] Torres VJ, Stauff DL, Pishchany G, Bezbradica JS, Gordy LE (2007). A Staphylococcus aureus regulatory system that responds to host heme and modulates virulence. Cell Host Microbe.

[21] Lechardeur D, Cesselin B, Liebl U, Vos MH, Fernandez A (2012). Discovery of intracellular heme-binding protein HrtR, which controls heme efflux by the conserved HrtB-HrtA transporter in *Lactococcus lactis*. J Biol Chem.

[22] Wakeman CA, Stauff DL, Zhang Y, Skaar EP (2014). Differential activation of *Staphylococcus aureus* heme detoxification machinery by heme analogues. J Bacteriol.

[23] Stauff DL, Skaar EP (2009). Bacillus anthracis HssRS signalling to HrtAB regulates haem resistance during infection. Mol Microbiol.

[24] Wakeman CA, Hammer ND, Stauff DL, Attia AS, Anzaldi LL (2012). Menaquinone biosynthesis potentiates haem toxicity in Staphylococcus aureus. Mol Microbiol.

[25] Edwards AM (2012). Phenotype switching is a natural consequence of Staphylococcus aureus replication. J Bacteriol.

[26] Duthie ES, Lorenz LL, coagulase S (1952). Mode of action and antigenicity. J Gen Microbiol.

[27] Attia AS, Benson MA, Stauff DL, Torres VJ, Skaar EP (2010). Membrane damage elicits an immunomodulatory program in *Staphylococcus aureus*. PLoS Pathog.

[28] Hammer ND, Reniere ML, Cassat JE, Zhang Y, Hirsch AO (2013). Two heme-dependent terminal oxidases power *Staphylococcus aureus* organ-specific colonization of the vertebrate host. mBio.

[29] Stauff DL, Bagaley D, Torres VJ, Joyce R, Anderson KL (2008). *Staphylococcus aureus* HrtA is an ATPase required for protection against heme toxicity and prevention of a transcriptional heme stress response. J Bacteriol.

[30] Aich A, Freundlich M, Vekilov PG (2015). The free heme concentration in healthy human erythrocytes. Blood Cells Mol Dis.

[31] Dean MA, Olsen RJ, Long SW, Rosato AE, Musser JM (2014). Identification of point mutations in clinical *Staphylococcus aureus* strains that produce small-colony variants auxotrophic for menadione. Infect Immun.

[32] Lechner S, Lewis K, Bertram R (2012). Staphylococcus aureus persisters tolerant to bactericidal antibiotics. J Mol Microbiol Biotechnol.

[33] Bisht K, Wakeman CA (2019). Discovery and therapeutic targeting of differentiated biofilm subpopulations. Front Microbiol.

[34] Amato SM, Fazen CH, Henry TC, Mok WWK, Orman MA (2014). The role of metabolism in bacterial persistence. Front Microbiol.

[35] Morikawa K, Ohniwa RL, Ohta T, Tanaka Y, Takeyasu K (2010). Adaptation beyond the stress response: cell structure dynamics and population heterogeneity in *Staphylococcus aureus*. Microbes Environ.

[36] Ayala OD, Wakeman CA, Pence IJ, Gaddy JA, Slaughter JC (2018). Drug-resistant *Staphylococcus aureus* strains reveal distinct biochemical features with Raman microspectroscopy. ACS Infect Dis.

